# Immune biomarkers of treatment failure for a patient on a phase I clinical trial of pembrolizumab plus radiotherapy

**DOI:** 10.1186/s13045-016-0328-4

**Published:** 2016-09-23

**Authors:** Gregory S. Alexander, Joshua D. Palmer, Madalina Tuluc, Jianqing Lin, Adam P. Dicker, Voichita Bar-Ad, Larry A Harshyne, Jennifer Louie, Colette M. Shaw, D. Craig Hooper, Bo Lu

**Affiliations:** 1 Sidney Kimmel Medical College at Thomas Jefferson University, Philadelphia, PA USA; 2 Department of Radiation Oncology, Bodine Center, Sidney Kimmel Medical College at Thomas Jefferson University, 111 South 11th Street, Philadelphia, PA 19107 USA; 3 Department of Pathology, Sidney Kimmel Medical College at Thomas Jefferson University, Philadelphia, PA USA; 4 Department of Medical Oncology, Sidney Kimmel Medical College at Thomas Jefferson University, Philadelphia, PA USA; 5 Department of Cancer Biology, Sidney Kimmel Medical College at Thomas Jefferson University, Philadelphia, PA USA; 6 Department of Interventional Radiology, Sidney Kimmel Medical College at Thomas Jefferson University, Philadelphia, PA USA

**Keywords:** Pembrolizumab, Renal cell carcinoma, Radiotherapy, Immunotherapy, Case report

## Abstract

**Background:**

Pembrolizumab is a monoclonal antibody that is designed against programmed cell death protein 1 (PD-1). Pembrolizumab and other immunocheckpoint-blocking monoclonal antibodies work by modulating a patient’s own immune system to increase anti-tumor activity. While immunocheckpoint blockade has shown promising results, only 20–40 % of patients experience objective clinical benefit. Differences in individual tumor biology and the presence multiple immune checkpoints present a challenge for treatment. Because radiotherapy has immunomodulatory effects on the tumor microenvironment, it has the potential to synergize with immunotherapy and augment tumor response. NCT02318771 is a phase 1 clinical trial designed to investigate the immunomodulatory effects of radiation therapy in combination with pembrolizumab.

**Case presentation:**

The patient is a 64-year-old male with metastatic clear cell renal cell carcinoma, Fuhrman grade 4, pathologically staged as T3 N0. Metastatic disease was well controlled for several years with sunitinib. Following disease progression, he was switched to axitinib. When disease progression continued, the patient was enrolled in NCT02318771, a phase 1 clinical trial combining radiotherapy and pembrolizumab. The patient experienced unusually rapid disease progression during treatment, which was confirmed by repeated CT scans to rule out pseudoprogression. Tissue biopsies and peripheral blood draws were obtained before, during, and after treatment. Samples were analyzed to provide plausible rationale for rapid treatment failure.

**Conclusions:**

Biomarker analysis demonstrated an absence of TILs, which may be a cause of treatment failure as pembrolizumab works through T cell-dependent mechanisms. Furthermore, the presence of other non-redundant immune checkpoints in the periphery and tumor microenvironment presents a treatment challenge. Additionally, the radiation dose and fractionation schedule may have played a role in treatment failure as these factors play a role in the effect radiotherapy on the tumor microenvironment as well as the potential for synergy with immunotherapy.

**Trial registration:**

An Exploratory Study to Investigate the Immunomodulatory Activity of Radiation Therapy (RT) in Combination With MK-3475 in Patients With Recurrent/Metastatic Head and Neck, Renal Cell Cancer, Melanoma and Lung Cancer, NCT02318771.

## Background

Pembrolizumab (MK-3475; Pembro) is a humanized IgG4 monoclonal antibody that is directed against the programmed cell death protein 1 (PD-1). PD-1 is expressed by T cells and interacts with its ligands programmed cell death protein ligand 1 (PD-L1) and programmed cell death protein ligand 2 (PD-L2), which are expressed in peripheral tissues and dendritic cells, respectively. PD-L1 expression is variable and involved in the prevention of autoimmunity; however, many tumors upregulate PD-L1 to mediate immune tolerance [1]. By blocking the PD-1 pathway, Pembro can enhance T cell-mediated killing of tumor cells [2]. It has been approved for the treatment of melanoma and non-small cell lung cancer; however, only 20–40 % of patients demonstrate clinical benefit with monotherapy [3–5]. Radiation therapy (RT) is thought to have immunostimulatory effects by enhancing cancer antigen presentation [6, 7]. Because both modalities work through immune-mediated mechanisms, combination therapy has the potential to augment immune activation by PD-1 checkpoint inhibitors. Preclinical models have demonstrated synergy between RT and PD-1 blockade treatments [8], and several clinical trials combining RT with immunotherapy are underway [9]. A phase 1 clinical trial (NCT02318771) was designed to investigate the immunomodulatory effects of RT/Pembro combination treatment.

We report clinicopathologic and detailed flow cytometry data from a patient with metastatic renal cell carcinoma (RCC) who progressed through combined immunotherapy and radiation.

## Case presentation

A 64-year-old Caucasian gentleman presented with flank pain and gross hematuria in February 2010. Initial work-up demonstrated a left renal mass, and a left radical nephrectomy confirmed clear cell RCC, Fuhrman grade 4, pathologically staged as T3 N0. Additionally, at diagnosis, small pulmonary nodules were found that were later confirmed to be RCC by Pax-8 staining (Fig. 3d). He was treated with sunitinib from March 2010 until July 2013, when disease progression occurred. The patient was then treated with axitinib, until progression and the development of additional pulmonary nodules in November 2014.

**Fig. 1:**
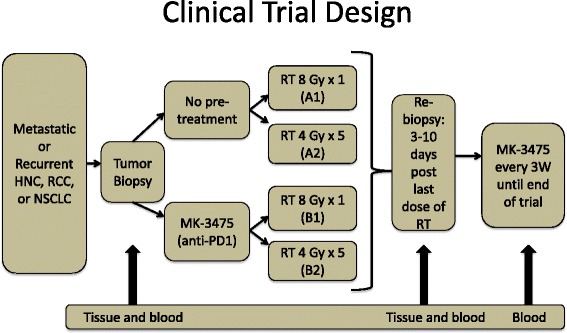
Clinical trial schema. Patients with metastatic or recurrent head and neck cancer, renal cell carcinoma, or non-small cell lung cancer were randomized to either receive pembrolizumab pre-treatment or no pembrolizumab pre-treatment. Patients were again randomized to receive a single dose of 8 Gy of radiation or five doses of 4 Gy of radiation. Tumor biopsies were obtained at the beginning of the trial as well as 3–10 days following the last dose of RT. Following RT, all patients received 200 mg of pembrolizumab every 3 weeks

**Fig. 2:**
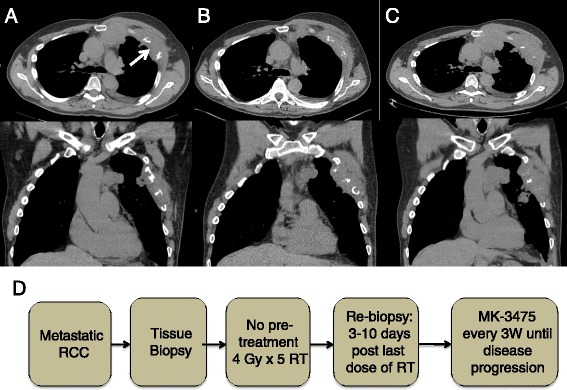
CT scans of the patient demonstrate disease progression. **a** Axial and coronal CT images taken before treatment. The *white arrow* indicates the target lesion, which was biopsied before and after RT. This lesion in the left anterior chest wall measured 20 × 13 mm. Other nodules included the right lower lobe lesion measuring 8 × 8 mm and the right lung base measuring 5 × 2 mm. **b** Axial and coronal CT images of patient 3 months post-treatment demonstrating disease progression. The left anterior chest wall mass increased from 20 × 13 mm to 24 × 21 mm. The right lower lobe nodule increased in size from 8 × 8 mm to 18 × 16 mm, and the right lung base increased from 5 × 2 mm to 11 × 13 mm. **c** Axial and coronal CT images 4 months post-treatment were performed to rule out pseudoprogression. True radiographic progression was confirmed. The left anterior chest wall mass increased from 24 × 21 mm to 30 × 29 mm. The right lower lobe nodule remained 18 × 16 mm, and the right lung base increased from 11 × 13 mm to 17 × 12 mm. **d** Schema displaying the patient’s course of treatment. The patient was randomized to group A2. He received no pembrolizumab pre-treatment, 20 Gy of radiation delivered over the course of five treatments followed by 200 mg of pembrolizumab every 3 weeks until disease progression

**Fig. 3:**
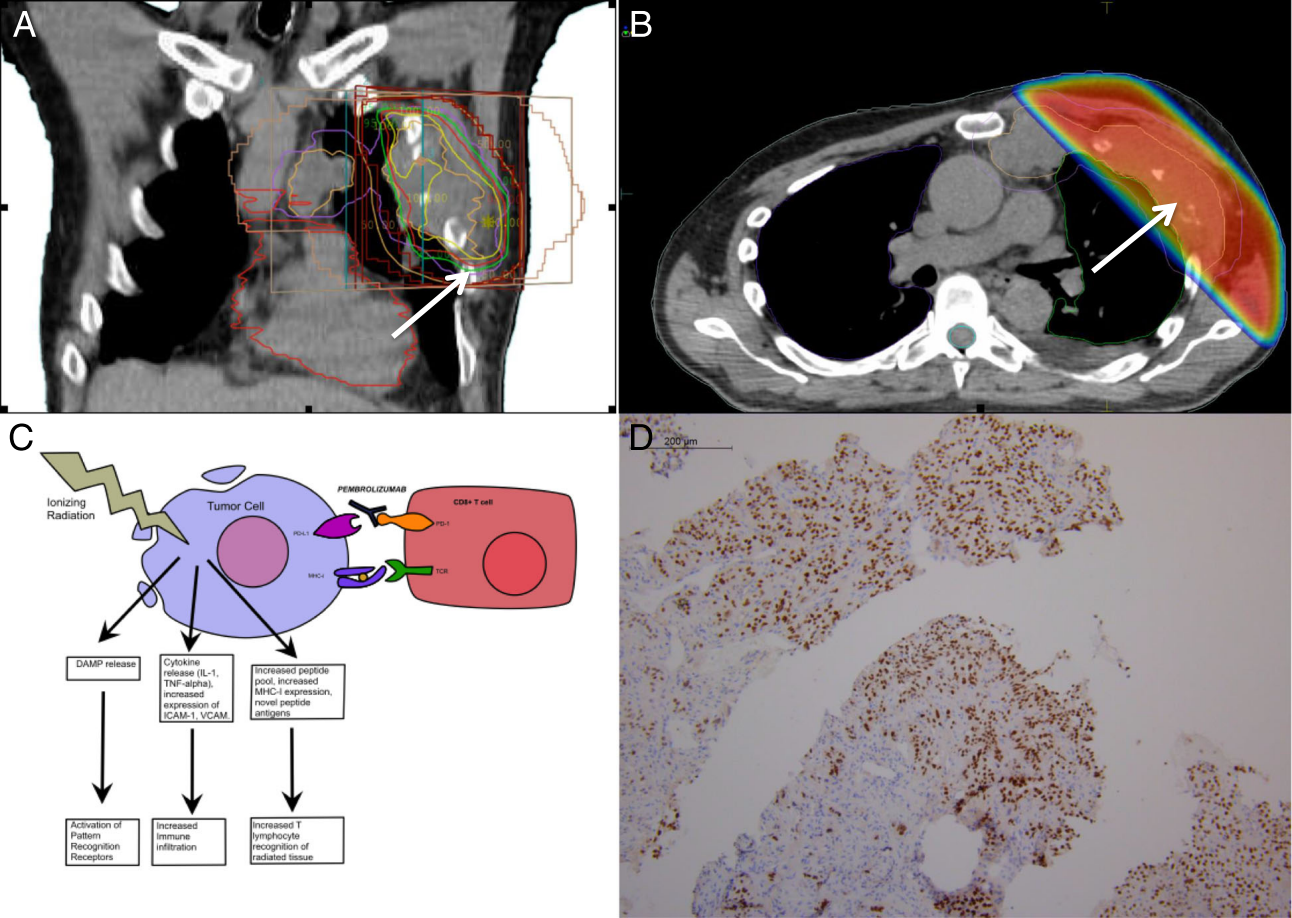
**a** Coronal image of the radiation treatment planning CT depicting the treatment portal, organs at risk, and isodose lines. The *white arrow* points to the 20-Gy isodose line. **b** Axial image of the radiation treatment planning CT depicting the dose wash and dose falloff into the left lung. *Red* depicts the prescription isodose line of 20 Gy, which was given over five daily fractions. The *white arrow* points to the 20-Gy isodose line. **c** The immunomodulatory effects of ionizing radiation has the potential for synergism with pembrolizumab. **d** A tumor biopsy of chest wall lesion was stained with Pax-8 to confirm metastatic RCC

The patient was thereafter enrolled in a clinical trial (NCT02318771). The trial schema is illustrated in Fig. 1. The trial enrolls patients with metastatic lung cancer, melanoma, head and neck cancer, or RCC. The primary objective is to determine immune activation from single dose 8 Gy vs. fractionated palliative radiotherapy (4 Gy ×5), which is either preceded by one cycle of Pembro or followed by Pembro that continues until disease progression or grade 3 or greater toxicities. Our patient was randomized to group A2, (Fig. 2d) in which he was given a course of palliative RT dose (20 Gy in five fractions to his left chest wall lesion), (Fig. 3a and b) followed by adjuvant Pembro 200 mg once every 3 weeks intravenously. After 5 cycles of treatment, CT scans in May 2015 (Fig. 2b) and a confirmatory scan in June 2015 (Fig. 2c) revealed progression when compared to his baseline scans taken in February 2015 (Fig. 2a). Patient tolerated the combined therapy well without any toxicities and had no complaints of chest wall or sternal discomfort or respiratory symptoms although imaging studies demonstrated an increased size of the left chest wall lesion, multiple bilateral pulmonary nodules, and an anterior mediastinal mass accompanied with erosion of the sternum and the third, fourth, and fifth ribs, as well as new nodules that were not previously identified. As per protocol, the patient was removed from trial following disease progression. A biopsy of the chest wall lesion was performed to rule out possible pseudoprogression.

**Fig. 4:**
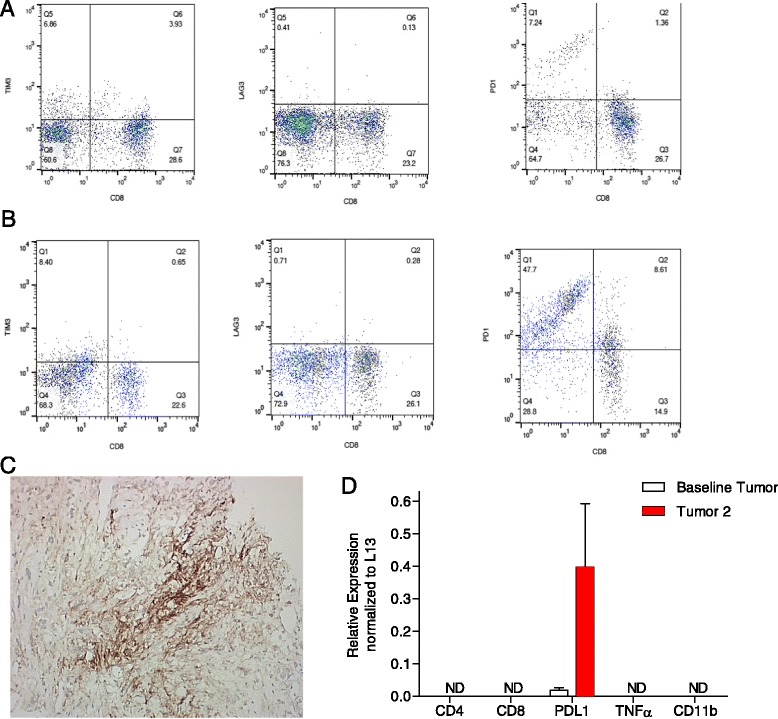
Flow cytometry analysis of peripheral blood T cells before (**a**) and after treatment (**b**). The expression of the immune markers (from *left* to *right*) TIM-3, Lag-3, and PD-1, on CD3^+^ T cells counter-stained for CD8 were analyzed. **c** Immunostaining of tumor cells following radiotherapy for PD-L1. **d** Real-time quantitative RT-PCR analysis of baseline and post radiotherapy tumor biopsy specimens for the content of mRNAs specific for the T cell markers CD4, CD8, the checkpoint inhibitor PD-L1, the pro-inflammatory cytokine TNFα, and the macrophage marker CD11b. *ND* none detected. The quantitative RT-PCR assay uses artificial gene standards for positive controls and is sensitive to the level of 10 copies per sample

Peripheral blood draws and tumor biopsies of his target lesion were obtained before, after RT in early March, and at the time of progression in late June. Flow cytometry analysis of his circulating CD3^+^ T lymphocytes revealed a minor change in the proportion of CD8+ lymphocytes from baseline (28 %) compared to post-radiation (24 %) and post-Pembro treatment (24 %). However, due to a decrease in the numbers of circulating CD3^+^ T cells from 1.2 × 10^6^/ml before study to 0.9 × 10^6^/ml at the conclusion of the trial, there was an overall reduction in the number of circulating CD8 T cells by 40 %. This was accompanied by a somewhat lesser 22 % decrease in the numbers of circulating CD4+ T cells post-Pembro. Over the course of treatment, PD1 expression was strongly increased on subsets of circulating CD8^+^ and CD8^−^ CD3^+^ T cells (Fig. 4a, b). Changes in the expression of other immune checkpoint markers including Tim-3 and Lag-3 were also assessed by flow cytometry. Both were detected on circulating CD8+ cells, but there were no significant differences in expression between cells obtained before and after treatment noted (Fig. 4a, b).

There was an absence of tumor-infiltrating lymphocytes (TILs) on tumor biopsy by histological analysis. This finding was confirmed by RT-PCR analyses of tumor biopsies, which failed to detect CD4 or CD8 transcripts (Fig. 4d). RT-PCR also failed to detect TNF-alpha or CD11b transcripts before and after radiotherapy. PD-L1 transcripts were strongly upregulated in biopsy tissue following RT, which was confirmed by immunohistochemistry (Fig. 4c).

### Discussion

The investigational agent Pembro works through antagonism of PD-1 pathway on activated T cells. While PD-1 blockade monotherapy has led to impressive results against some treatment resistant tumors [10], single checkpoint blockade only produces an objective clinical response in approximately 20–40 % of patients [3–5]. It appears that PD-L1 expression may be predictive of treatment response, but not all patients with PD-L1-positive tumors respond [11]. Because there are multiple, non-redundant mediators of immune tolerance in the microenvironment, tumors may escape immune surveillance through other pathways that are not disrupted by Pembro [1].

Activated T cells may express multiple immunomodulatory cell surface receptors, such as TIM-3, LAG-3, and CTLA-4 [12]. When these receptors are engaged by their respective ligands, T cell activity is repressed. Flow cytometry of peripheral blood mononuclear cells revealed TIM-3 and Lag-3 expression on circulating T lymphocytes, which suggests multiple pathways may have been utilized by the metastatic growths to evade the immune response. Preclinical animal models have demonstrated increased efficacy when various combinations of different immune checkpoint blockade and RT are utilized when compared to monotherapy [8, 13–16].

Of note is the lack of TILs, which was confirmed by RT-PCR and histological study and the 40 % decrease in total circulating CD8-positive T cells. Because Pembro acts through a T cell-dependent mechanism, a decrease in circulating CD8s and a lack of TILs is a possible cause for treatment failure [17]. The presence of TILs has been shown to be predictive treatment response in both melanoma and mismatch repair-deficient colon cancer [18, 19], but more work must be done to demonstrate predictive value in other solid tumors [20]. The lack of TILs in this patient may be attributed to a dysregulation of chemokine secretion, resulting in a failure to attract migrating lymphocytes into target tissue. This hypothesis is supported by the failure of RT-PCR to detect TNF-alpha transcripts in tumor biopsies before and after treatment as TNF-alpha has a role in lymphocytic migration. The lengthy treatment with tyrosine kinase inhibitors, axitinib, and sunitinib, may have also inhibited lymphocytic infiltration. Both drugs have been shown to have immunomodulatory effects that can disrupt the function of both T cells and dendritic cells [21–24].

In addition to inducing apoptosis through free radical damage, RT has the potential to enhance T cell recognition of malignant tissue through the induction of MHC-I expression and generation of neoantigens [25]. These alterations in the immune microenvironment can result in regression of the target lesion, as well as distant metastases. Reduction in the size of non-irradiated lesions following RT is a rare phenomenon called the abscopal effect, which is hypothesized to be mediated through the increased immune activation that can result following RT [26]. The abscopal effect has been reported in several cases of RCC [27]. It can result in dramatic clinical improvement, and there has been some success in recreating it in clinical trials [28]. Because this patient was unable to generate a sufficient immune response, anti-tumor activity was observed in neither distant nor target lesions.

The primary end point of our trial is to explore the immune modulating effects of RT such as upregulation of PDL1 in several cancer types. While this clinical trial is still ongoing, PD-L1 transcripts in this patient’s tumor biopsy tissue were greatly increased following RT. Other studies have found PD-L1 to be upregulated following RT and that increased anti-tumor activity was observed by combining RT with PD-L1 blockade [29]. Importantly, this benefit was only observed when concurrent treatment was delivered and not when RT was delivered prior to PD-L1 blockade [30]. Our patient was randomized to receive Pembro following RT, which may have made treatment failure more likely.

To further complicate issues, the dosage and method of delivery of RT is an important factor in treatment outcome as preclinical models have shown that RT can either stimulate or repress the immune system based on delivery and dosage [31]. A preclinical study demonstrated that the abscopal effect and synergism with an anti-CTLA-4 monoclonal antibody was seen only when RT was hypofractionated and not with other RT fractionations [32]. There is also risk of immunosuppression as RT can increase the proportion of immunosuppressive T regulatory cells (Treg) [33]. Additionally, RT may exacerbate pre-existing M2 macrophage polarization in the tumor microenvironment, decrease the CD8 to Treg ratio, and induce apoptosis of TILs [34]. The optimal dose and delivery needed to optimize therapy may also be dependent on the biology of each individual tumor and therefore requires further study.

There are many ongoing attempts to use various combinations of immune checkpoint blockades and RT to maximize anti-tumor activity [35, 36]. Results so far have been encouraging. Patient subset analysis has found that RT/ipilimumab treatment-resistant melanoma patients with high PD-L1 expression benefit from PD-L1 blockade [13]. Additionally, a clinical trial combining nivolumab and ipilimumab demonstrated an improved clinical response in melanoma patients when compared to monotherapy, with some evidence suggesting PD-L1 positivity is predictive of longer progression free survival [37, 38]. However, the safety profile of combination therapy is unknown and optimization requires careful consideration.

## Conclusions

In conclusion, we hypothesize that the failure of T lymphocytes to migrate into malignant tissue may have played a role in treatment failure as both RT and Pembro act through T cell-dependent mechanisms. Further optimization of combined RT and immunotherapy requires a more in-depth understanding of the tumor microenvironment. In order to maximize anti-tumor activity, the presence of multiple, non-redundant checkpoint regulators, as well as the dose- and schedule-dependent immunomodulatory effects of RT, must be taken into consideration.

## Abbreviations

Pembro: Pembrolizumab
PD-1: Programmed cell death protein 1
PD-L1: Programmed cell death protein ligand 1
PD-L2: Programmed cell death protein ligand 2
RT: Radiation therapy
RCC: Renal cell carcinoma
TILs: Tumor-infiltrating lymphocytes
Tregs: T regulatory cells

## References

[1] Pardoll DM (2012). The blockade of immune checkpoints in cancer immunotherapy. Nat Rev Cancer.

[2] Dolan DE, Gupta S (2014). PD-1 pathway inhibitors: changing the landscape of cancer immunotherapy. Cancer Control J Moffitt Cancer Cent.

[3] Rizvi NA, Mazières J, Planchard D, Stinchcombe TE, Dy GK, Antonia SJ (2015). Activity and safety of nivolumab, an anti-PD-1 immune checkpoint inhibitor, for patients with advanced, refractory squamous non-small-cell lung cancer (CheckMate 063): a phase 2, single-arm trial. Lancet Oncol.

[4] Swaika A, Hammond WA, Joseph RW (2015). Current state of anti-PD-L1 and anti-PD-1 agents in cancer therapy. Mol Immunol.

[5] Massari F, Santoni M, Ciccarese C, Santini D, Alfieri S, Martignoni G (2015). PD-1 blockade therapy in renal cell carcinoma: current studies and future promises. Cancer Treat Rev.

[6] Formenti SC, Demaria S (2009). Systemic effects of local radiotherapy. Lancet Oncol.

[7] Reits EA, Hodge JW, Herberts CA, Groothuis TA, Chakraborty M, Wansley EK (2006). Radiation modulates the peptide repertoire, enhances MHC class I expression, and induces successful antitumor immunotherapy. J Exp Med.

[8] Zeng J, See AP, Phallen J, Jackson CM, Belcaid Z, Ruzevick J (2013). Anti-PD-1 blockade and stereotactic radiation produce long-term survival in mice with intracranial gliomas. Int J Radiat Oncol Biol Phys.

[9] Daly ME, Monjazeb AM, Kelly K (2015). Clinical trials integrating immunotherapy and radiation for non-small-cell lung cancer. J Thorac Oncol Off Publ Int Assoc Study Lung Cancer.

[10] Davar D, Socinski MA, Dacic S, Burns TF (2015). Near complete response after single dose of nivolumab in patient with advanced heavily pre-treated KRAS mutant pulmonary adenocarcinoma. Exp Hematol Oncol.

[11] Smith AD, Roda D, Yap TA (2014). Strategies for modern biomarker and drug development in oncology. J Hematol Oncol.

[12] Nirschl CJ, Drake CG (2013). Molecular pathways: coexpression of immune checkpoint molecules: signaling pathways and implications for cancer immunotherapy. Clin Cancer Res Off J Am Assoc Cancer Res.

[13] Twyman-Saint Victor C, Rech AJ, Maity A, Rengan R, Pauken KE, Stelekati E (2015). Radiation and dual checkpoint blockade activate non-redundant immune mechanisms in cancer. Nature.

[14] Duraiswamy J, Kaluza KM, Freeman GJ, Coukos G (2013). Dual blockade of PD-1 and CTLA-4 combined with tumor vaccine effectively restores T cell rejection function in tumors. Cancer Res.

[15] Jing W, Gershan JA, Weber J, Tlomak D, McOlash L, Sabatos-Peyton C (2015). Combined immune checkpoint protein blockade and low dose whole body irradiation as immunotherapy for myeloma. J Immunother Cancer.

[16] Woo S-R, Turnis ME, Goldberg MV, Bankoti J, Selby M, Nirschl CJ (2012). Immune inhibitory molecules LAG-3 and PD-1 synergistically regulate T-cell function to promote tumoral immune escape. Cancer Res.

[17] Ascierto PA, Kalos M, Schaer DA, Callahan MK, Wolchok JD (2013). Biomarkers for immunostimulatory monoclonal antibodies in combination strategies for melanoma and other tumor types. Clin Cancer Res Off J Am Assoc Cancer Res.

[18] Tumeh PC, Harview CL, Yearley JH, Shintaku IP, Taylor EJM, Robert L (2014). PD-1 blockade induces responses by inhibiting adaptive immune resistance. Nature.

[19] Le DT, Uram JN, Wang H, Bartlett BR, Kemberling H, Eyring AD (2015). PD-1 blockade in tumors with mismatch-repair deficiency. N Engl J Med.

[20] Schalper KA, Kaftan E, Herbst RS (2016). Predictive biomarkers for PD-1 axis therapies: the hidden treasure or a call for research. Am Assoc Cancer Res.

[21] Gu Y, Zhao W, Meng F, Qu B, Zhu X, Sun Y (2010). Sunitinib impairs the proliferation and function of human peripheral T cell and prevents T-cell-mediated immune response in mice. Clin Immunol Orlando Fla.

[22] Jaini R, Rayman P, Cohen PA, Finke JH, Tuohy VK (2014). Combination of Sunitinib with anti-tumor vaccination inhibits T cell priming and requires careful scheduling to achieve productive immunotherapy. Int J Cancer J Int Cancer.

[23] Stehle F, Schulz K, Fahldieck C, Kalich J, Lichtenfels R, Riemann D (2013). Reduced immunosuppressive properties of axitinib in comparison with other tyrosine kinase inhibitors. J Biol Chem.

[24] Heine A, Held SAE, Daecke SN, Riethausen K, Kotthoff P, Flores C, et al. The VEGF-receptor inhibitor axitinib impairs dendritic cell phenotype and function. PLoS ONE [Internet]. 2015 [cited 2015 Dec 22];10. Available from: http://www.ncbi.nlm.nih.gov/pmc/articles/PMC4456373/. 10.1371/journal.pone.0128897 PMC4456373 26042424

[25] Corso CD, Ali AN, Diaz R (2011). Radiation-induced tumor neoantigens: imaging and therapeutic implications. Am J Cancer Res.

[26] Reynders K, Illidge T, Siva S, Chang JY, De Ruysscher D (2015). The abscopal effect of local radiotherapy: using immunotherapy to make a rare event clinically relevant. Cancer Treat Rev.

[27] Wersäll PJ, Blomgren H, Pisa P, Lax I, Kälkner K-M, Svedman C (2006). Regression of non-irradiated metastases after extracranial stereotactic radiotherapy in metastatic renal cell carcinoma. Acta Oncol Stockh Swed.

[28] Golden EB, Chhabra A, Chachoua A, Adams S, Donach M, Fenton-Kerimian M (2015). Local radiotherapy and granulocyte-macrophage colony-stimulating factor to generate abscopal responses in patients with metastatic solid tumours: a proof-of-principle trial. Lancet Oncol.

[29] Dovedi SJ, Adlard AL, Lipowska-Bhalla G, McKenna C, Jones S, Cheadle EJ (2014). Acquired resistance to fractionated radiotherapy can be overcome by concurrent PD-L1 blockade. Cancer Res.

[30] Dovedi SJ, Illidge TM (2015). The antitumor immune response generated by fractionated radiation therapy may be limited by tumor cell adaptive resistance and can be circumvented by PD-L1 blockade. Oncoimmunology.

[31] Schaue D, Ratikan JA, Iwamoto KS, McBride WH (2012). Maximizing tumor immunity with fractionated radiation. Int J Radiat Oncol Biol Phys.

[32] Dewan MZ, Galloway AE, Kawashima N, Dewyngaert JK, Babb JS, Formenti SC (2009). Fractionated but not single-dose radiotherapy induces an immune-mediated abscopal effect when combined with anti-CTLA-4 antibody. Clin Cancer Res Off J Am Assoc Cancer Res.

[33] Persa E, Balogh A, Sáfrány G, Lumniczky K (2015). The effect of ionizing radiation on regulatory T cells in health and disease. Cancer Lett.

[34] Schaue D, Micewicz ED, Ratikan JA, Xie MW, Cheng G, McBride WH (2015). Radiation and inflammation. Semin Radiat Oncol.

[35] Salama AKS, Postow MA, Salama JK (2016). Irradiation and immunotherapy: from concept to the clinic. Cancer.

[36] Vilgelm AE, Johnson DB, Richmond A (2016). Combinatorial approach to cancer immunotherapy: strength in numbers. J Leukoc Biol.

[37] Postow MA, Chesney J, Pavlick AC, Robert C, Grossmann K, McDermott D (2015). Nivolumab and ipilimumab versus ipilimumab in untreated melanoma. N Engl J Med.

[38] Tsai KK, Daud AI. Nivolumab plus ipilimumab in the treatment of advanced melanoma. J Hematol Oncol. [Internet]. 2015 [cited 2016 Aug 20];8. Available from: http://www.ncbi.nlm.nih.gov/pmc/articles/PMC4628394/. 10.1186/s13045-015-0219-0 PMC4628394 26518223

